# Editorial: Prey-predator interactions

**DOI:** 10.3389/fnbeh.2024.1367484

**Published:** 2024-02-08

**Authors:** F. C. Rind, Vincent Bels

**Affiliations:** ^1^Centre for Behaviour and Evolution, Biosciences Institute, Newcastle University, Newcastle upon Tyne, United Kingdom; ^2^Muséum national d'Histoire naturelle, Sorbonne Université, Institut de Systématique, Evolution, Biodiversité, UMR 7205 CNRS/MNHN, Paris, France

**Keywords:** snake, crab, squid, binocular-vision, escape, capture

The scope of this Research Topic: prey-predator interactions is deliberately broad to encourage examples from diverse perspectives. Prey-predator interactions are one of the key pressures explaining the evolution and adaptation of many traits in organisms, from microorganisms to vertebrates. These predator-prey interactions influence fitness at different biological levels, from individuals to community structures and dynamics of populations. In a classical view, these interactions elicit mutual adaptations that improve predator success, traits such as morphology (e.g., wings, claws, teeth), physiology (e.g., sensory processing, speed, acceleration, maneuverability) as well as prey traits (e.g., predator detection, antipredator behavior, morphological and chemical repulsion, crypsis, aposematism, mimicry), Improvements occur at the individual level, and the intra- and interspecific group level (e.g., hunting strategy in a predator, the collective response of prey). From the point of view of both the predator and the prey, such adaptive responses to natural stimuli are under complex neuronal and hormonal control. The aim of this Research Topic on prey-predator interactions is to describe advances at different levels (e.g., descriptive, conceptual, modeling) from the predator and the prey's points of view and sometimes both. Articles concern behaviors (e.g., foraging, detection, feeding, prey/food consuming) of phylogenetically diverse predators: fish, red knot birds, rats, mice, natricine snakes, geckos, semiterrestrial crabs squid and of their prey (e.g., detection, defense, escape).

The first article—*Incorporating neurological and behavioural mechanisms of sociality into predator-prey models*—explores the role of social context in modeling predators at the population level (Lichtenstein and Schmitz). Whereas previously, population level consumption-resource had been simply extrapolated linearly, from a measure of consumption-resource of individual predators, assuming, that individuals forage independently of each other. Lichtenstein and Schmitz give several examples that contradict this assumption. Examples such as: position of the predator in a dominance hierarchy; sociality in predator species; or traits, such as short-term aggressiveness measured in individual fish that positively affect predation success and weight-gain in the short term and in the long-term as aggressiveness of some individuals in a population persists through life. Each gives an example where behavior can cause consumer-resource interactions between individuals. The article goes on to suggest how to incorporate interactions into a population resource model of predation.

*Using natricine snakes to test how prey type and size affect predatory behaviours and performance*—All snakes are predators which swallow prey whole (Gripshover and Jayne). *Liodytes rigida* and *Liodytes pygaea*, two species of small natricine snakes, in the clade *Liodytes*, are specialist crayfish eaters. To determine whether apparent behavioral stereotypy of food handling in natricine snakes is due to the narrow range of stimuli used to study it, feeding behavior was compared when eating different sizes of small crayfish, *Orconectes rusticus*, at different stages in their molting cycle. Crayfish are defended by a hardened exoskeleton, except when molting. The snakes used a range of techniques for prey capture. Only *L. rigida* used envenomation, particularly with hardened crayfish. Videos of crayfish capture by *L. rigida* illustrate the speed and flexibility of this snake's predatory behavior. *Liodytes alleni* also in the *Liodytes* clade and a sister species to *L. rigida*, were given their traditional vertebrate prey (juvenile *Siren intermedia* salamanders or mosquito fish) to find if some of the behaviors that facilitate eating crayfish were present before the transition from vertebrate to invertebrate prey. Even with vertebrate prey, *L. pygaea* never used coiling or envenomation, whereas previous studies of *L. alleni*, the sister species of *L. rigida*, observed non-lethal coiling without envenomation when eating hard-shell crayfish. For the *Liodytes* clade of three species, this implies that coiling evolved ancestral to the two crayfish specialists (*L. alleni; L. rigida*). Envenomation by *L. rigida* subsequently evolved as an additional means of subduing formidable prey. The proximate benefits observed for coiling and envenomation in *L. rigida* support the evolutionary scenario that both traits enhanced feeding performance for more formidable prey.

*The tailless gecko gets the worm: prey type alters the effect of caudal autotomy on prey capture and subjugation kinematics***—**Some prey items are easier to handle than others for a tailless gecko (Vollin and Higham). Using high-speed 3D videography, Vollin and Higham studied the effects of both prey type (mealworms and crickets) and tail autotomy on prey capture and subjugation performance in banded geckos. Whereas crickets are able to evade capture, mealworms are not. Performance metrics included maximum velocity and distance between predator and the prey captured, as well as velocity and frequency of post-capture shaking. Maximum velocity and distance of prey capture were lower for mealworms than crickets regardless of tail state. However, after autotomy, maximum velocity increased for strikes on mealworms but significantly decreased for crickets. Tail state did not significantly affect the percentage of successful strikes for cricket (Wilcoxon signed-rank test, *z* = 7, *P* = 1) or mealworm trials. After capture, geckos always shook mealworms, but never crickets. The frequency of shaking mealworms decreased after autotomy but the vigor with which the gecko shook its mealworm prey increased and in some cases the geckos' feet were lifted off the substrate with the gecko becoming airborne. In natural populations 74% of adults had either missing or regenerated tails. The results highlight the complex and interactive effects of prey type (evasive vs. non-evasive) and caudal autotomy on prey capture biomechanics. Prey capture and handling reach their pre-autotomy levels after tail loss. Adult nervous system reorganization could account for the change in motor behaviors, like shaking strength, and in the perceptual decisions recognizing prey types. The reorganization of the adult nervous system after tail loss makes the gecko a potential model for studying adult nervous system plasticity.

Predation events can generally be divided into five main phases: encounter, detection, pursuit, subjugation and consumption [Endler, 1986; cited in Downes and Shine (2001)], with prey capture referring specifically to events that take place during pursuit and subjugation. Lizards have become a model system for prey capture studies (reviewed in Schwenk, 2000; Bels et al., 2019; Montuelle and Kane, 2019), with most studies focusing on how the prey is captured with the jaws or tongue. Measurements within these studies focus on the cranial movements of the skull (Montuelle et al., 2012), but post-cranial movements have received far less attention, despite the clear dependence on locomotor systems in capturing elusive prey (Bels et al., 2019).

*Predatory behavior under monocular and binocular conditions in the semiterrestrial crab Neohelice granulate*—*Neohelice granulata* crabs live in mudflats where they prey upon smaller crabs (Harper et al.). Predatory behavior can be elicited in the laboratory by a dummy moving at ground level in an artificial arena. To estimate the distance to an object on the ground, *Neohelice* could rely on angular declination below the horizon or, since they are broad fronted with eye stalks far apart, on stereopsis. The authors observed a strong reduction in the probability of predation in monocular crabs, with one eye covered, accompanied by a rise in the probability of not responding or actively freezing. Additionally, several characteristics of the predatory responses had changed. The current study indicates that for taking the decision to initiate a predatory behavior the availability of both eyes is extremely important in crabs. The presence of binocular cues also improved the proportion of complete and successful attacks. Yet, a definite proof of the use of stereopsis in crabs is still pending. Establishing the use of stereopsis is challenging and so far, only two invertebrates have been conclusively added to the list of animals able to estimate distance by stereopsis, the praying mantis (Maldonado and Rodriguez, 1972; Rossel, 1986; Nityananda et al., 2016) and the cuttlefish (Feord et al., 2020). In both cases, the ultimate proof has been achieved by modifying the visual perception of the animal with anaglyph 3D images and color filter lenses while measuring the distance of the ballistic attacks produced. In praying mantis, neurons proposed to be involved in the neural network mediating stereopsis have been found recently (Rosner et al., 2017). Neurons with similar properties have been described in damselflies (Supple et al., 2020) and in *Neohelice granulata* crabs (Scarano et al., 2018) providing strong candidates for animals that use stereopsis. The range of depth estimation is limited by the interocular distance, which is quite small in most insects (Olberg et al., 2005) but is considerably broader in the case of *N. granulata*. Theoretical calculations are described that suggest this crab would be able to estimate distances up to 180 cm. The confirmation of stereopsis in a crab awaits further research but is more likely after the research detailed here.

*Predicting the effects of spatiotemporal modifications of muscle activation on the tentacle extension in squid—*Relating muscular action to prey capture in animals without a ridged skeleton is a rapidly growing research area (van Leeuwen and Kier). Squid use eight arms and two slender tentacles to capture prey. High-speed cinematography of prey capture by the squid *Doryteuthis pealeii* (formerly *Loligo pealeii*) reveals that the stalks elongate by ~50%−80% in only 20–40 ms, reaching peak velocities of over 2 m s^−1^ and peak accelerations of ~250 m s^−2^ (25.5 g) (Kier and Leeuwen, 1997). The authors predict how spatial muscle-activation patterns result in a distribution of muscular power, muscle work, and kinetic and elastic energy along the tentacle, using an existing model that describes the extension of the tentacles of the squid *D. pealeii*. The authors discovered that the simulated peak extension speed of the tentacles was stable and was insensitive to delays of activation along the stalk, as well as to random variations in the activation onset. A delay along the tentacle of 50% of the extension time had only a small effect on the peak extension velocity of the tentacle compared with a zero-delay pattern. A slight delay of the distal portion relative to the proximal has a small positive effect on peak extension velocity, whereas negative delays (delay reversed along stalk) always reduce extension performance. In addition, tentacular extension was relatively insensitive to superimposed random variations in the prescribed delays along the stalk. This held for small positive delays that are like delays predicted from measured axonal diameters of motor neurons. This robustness against variation in the activation distribution reduces the accuracy requirements of the neuronal control and is likely due to the non-linear mechanical properties of the muscular tissue in the tentacle. The tolerance of squid tentacle peak extension to timing of muscular contraction is a useful property that suits body and nervous system control: action and actuator.

## Author contributions

FR: Writing – original draft. VB: Conceptualization, Writing – review & editing.
